# Value and impact factors of multidetector computed tomography in diagnosis of preoperative lymph node metastasis in gastric cancer: A PRISMA-compliant systematic review and meta-analysis

**DOI:** 10.1097/MD.0000000000007769

**Published:** 2017-08-18

**Authors:** Mingxu Luo, You Lv, Xiuyu Guo, Hongmei Song, Guoqiang Su, Bo Chen

**Affiliations:** a Department of Gastrointestinal Surgery, Xiamen Cancer Hospital, The First Affiliated Hospital of Xiamen University, Xiamen, Fujian; b Department of Radiology, Xiamen Cancer Hospital, The First Affiliated Hospital of Xiamen University, Xiamen, Fujian; c Department of Oncology, Renmin Hospital of Shiyan, Hubei University of Medicine, Shiyan, Hubei, China.

**Keywords:** gastric cancer, lymph node metastasis, multidetector computed tomography, serosal invasion

## Abstract

**Background:**

Multidetector computed tomography (MDCT) exhibited wide ranges of sensitivities and specificities for lymph node assessment of gastric cancer (GC) in several individual studies. This present meta-analysis was carried out to evaluate the value of MDCT in diagnosis of preoperative lymph node metastasis (LNM) and to explore the impact factors that might explain the heterogeneity of its diagnostic accuracy in GC.

**Methods:**

A comprehensive search was conducted to collect all the relevant studies about the value of MDCT in assessing LNM of GC within the PubMed, Cochrane library and Embase databases up to Feb 2, 2016. Two investigators independently screened the studies, extracted data, and evaluated the quality of included studies. The sensitivity, specificity, and area under ROC curve (AUC) were pooled to estimate the overall accuracy of MDCT. Meta-regression and subgroup analysis were carried out to identify the possible factors influencing the heterogeneity of the accuracy.

**Results:**

A total of 27 studies with 6519 subjects were finally included. Overall, the pooled sensitivity, specificity, and AUC were 0.67 (95% CI: 0.56–0.77), 0.86 (95% CI: 0.81–0.90), and 0.86 (95% CI: 0.83–0.89), respectively. Meta-regression revealed that MDCT section thickness, proportion of serosal invasion, and publication year were the main significant impact factors in sensitivity, and MDCT section thickness, multiplanar reformation (MPR), and reference standard were the main significant impact factors in specificity. After the included studies were divided into 2 groups (Group A: studies with proportion of serosa-invasive GC subjects ≥50%; Group B: studies with proportion of serosa-invasive GC subjects <50%), the pooled sensitivity in Group A was significantly higher than in Group B (0.84 [95% CI: 0.75–0.90] vs 0.55 [95% CI: 0.41–0.68], *P* < .01). For early gastric cancer (EGC), the pooled sensitivity, specificity, and AUC were 0.34 (95% CI: 0.15–0.61), 0.91 (95% CI: 0.84–0.95), and 0.83 (95% CI: 0.80–0.86), respectively.

**Conclusion:**

To summarize, MDCT tends to be adequate to assess preoperative LNM in serosa-invasive GC, but insufficient for non-serosa-invasive GC (particularly for EGC) owing to its low sensitivity. Proportion of serosa-invasive GC subjects, MDCT section thickness, MPR, and reference standard are the main factors influencing its diagnostic accuracy.

## 1 Introduction

Despite a decrease in incidence over the past decades, gastric cancer (GC) remains one of the most common causes of cancer-related deaths worldwide.^[1]^ Radical surgery is the main effective intervention for cure or long-term survival.^[2]^ However, with new therapeutic options, such as endoscopic submucosal dissection and neoadjuvant chemotherapy, being introduced, accurate preoperative staging for GC is increasingly indispensable.^[3–5]^ Lymph node assessment is crucial to treatment strategy and determining prognosis in GC patients. In cases without distant metastases, extended lymphadenectomy based on the precise lymph node staging has been regarded as an important role of radical gastrectomy, which might improve the prognosis for GC.^[6,7]^ According to Japanese Gastric Cancer Association (JGCA), for differentiated T1a early gastric cancer (EGC) without lymph node metastasis (LNM), endoscopic resection or partial resection plus D1/D1+ lymphadenectomy should be considered, but patients with LNM need standard D2 lymphadenectomy.^[2]^ Besides, the occurrence of distant lymph node metastasis, which is classified as M1 staging, makes it impossible to operate with curative intent in patients with GC. In these cases, chemoradiotherapy and palliative surgery should be proposed.^[2]^ So having a good knowledge of preoperative lymph node assessment is of vital importance to make optimal treatment choice in patients with GC.^[8]^

Continuing evolutions in technology have made multidetector computed tomography (MDCT) become one of most common imaging modalities for GC staging prior to surgery.^[9,10]^ It was good for widely evaluating distant metastatic diseases, especially hepatic metastases, ascites, and distant nodal spread.^[11]^ However, the diagnostic accuracy of MDCT for assessing lymph node staging was inconsistent.^[10,12,13]^ In 2009, Kwee et al^[14]^ reported that the sensitivity and specificity varied from 62.5% to 91.9% (median: 80%) and from 50% to 87.9% (median: 77.8%), respectively. Although Seevaratnam et al^[15]^ had estimated its diagnostic performance of LNM by meta-analysis, rigorous inclusion criteria and quality assessment were absent and only 2 impact factors were discussed, which made their conclusion limited. Wang et al^[16]^ also conducted a meta-analysis about this topic, but they did not identify any impact factors contributing to the heterogeneity of their results. Besides, whether its accuracy was improved by technical development of isotropic imaging or multiplanar reformation (MPR) was still uncertain.^[17–19]^ Therefore, we constructed a meta-analysis to confirm whether the presence of preoperative LNM was reliably evaluated in GC by MDCT and to exhibit the possible factors influencing its diagnostic accuracy.

## 2 Materials and methods

### 2.1 Inclusion and exclusion criteria

Inclusion criteria for this meta-analysis were studies investigating the diagnostic performance of MDCT (defined as CT with 4 or more detectors) in predicting LNM in GC subjects. The participants clinically suspected of GC and diagnosed with GC by postoperative pathology were recruited; the diagnosis of positive lymph node (N+) was based on pathology after surgery; true-positive, false-positive, true-negative, and false-negative results of MDCT were available or allowed for calculation from original articles; for eligible studies with data published more than once: we only included the studies with the largest sample size of subjects. Exclusion criteria were studies that included subjects with non-primary GC; studies that included subjects who received preoperative radiotherapy or chemotherapy, which might cause tumor down-staging; case reports, review articles, in vitro studies, and animal experiments for GC; and studies with sample size <40.

### 2.2 Literature search

A comprehensive computer-aided literature search of PubMed, Cochrane library, and Embase databases was carried out to find relevant publications concerning the diagnostic value of MDCT in predicting preoperative LNM in GC subjects. We used a search algorithm based on a combination of the terms: “stomach cancer” or “gastric cancer” or “stomach carcinoma” or “gastric carcinoma” or “GC”; and “lymph node metastasis” or “nodal metastases” or “lymphatic metastasis” or “lymph node involvement” or “nodal involvement” or “lymph node status” or “lymph node staging” or “N staging” or “TNM”; and “computed tomography” or “CT” or “MDCT” or “multidetector computed tomography.” The search was performed from inception to February 2, 2016 and had no language restrictions. To expand our search coverage, the listed references of these retrieved articles were also manually screened for additional studies.

### 2.3 Study selection and data extraction

Two investigators (ML and XG) independently reviewed titles and abstracts of the retrieved articles, according to the aforementioned selection criteria. Articles were excluded if clearly ineligible. Then the full-text version of the selected articles was evaluated to determine their eligibility for inclusion. Finally, the aforementioned 2 reviewers cross-checked each independent selected study. Any controversy was resolved by consulting a third author (YL) and reconfirming whether the study was in strict accordance with the inclusion criteria. For each eligible study, the baseline information and data extraction were done independently by ML and XG. Then the 2 authors reached an agreement by cross-checking the information and extracted data. If there was any discrepancy, the aforementioned 2 reviewers would review the raw data of the included studies and have a discussion on the underlying causes of the objection, on the appropriate scope of application of the extracted data, and on whether the extraction was reliable. Then the more credible raw data was selected to reach a consensus. If an agreement was still unfinished, the third investigator (YL) would be involved to verdict the dissent.

The methodological quality was assessed according to a checklist adapted by Kelly et al^[20]^ and Kwee et al.^[14]^ This tool consisted of 13 question items with responses given as “yes,” “no,” or “not available.” If the response was “yes,” then the score of 1 was given, and if the response was “no” or “unavailable,” then the score of zero was given. From the 13 items, the aggregate score ≥8 was regarded as high quality and the aggregate score <8 was regarded as low quality.

### 2.4 Statistical analysis

The sensitivity and specificity were calculated for each study on a per-patient based analysis. A summary receiver operating characteristics (sROC) curve was constructed for recruited studies and area under ROC curve (AUC) was calculated to estimate the overall accuracy. A preferred test has an AUC close to 1, while a poor test has an AUC close to 0.5.

Study heterogeneity among those eligible studies was assessed by *I*^2^ test, with *I*^2^ >50% suggesting mild heterogeneity among studies. Threshold effect was an important extra source of variation in meta-analysis. To assess whether the threshold effect existed, the Spearman correlation test and bivariate boxplot were utilized to verify it.^[21]^ The bivariate boxplot describes the degree of interdependence including the central location and identification of any outliers. The inner oval (also known as bag) represents the median distribution of the data points and the outer oval (also known as fence) represents the 95% confidence bound. The points outside the fence are flagged as outliers, thus providing indirect evidence of some threshold variability in these studies.^[22]^

If study heterogeneity exists among those recruited studies, the potential sources of heterogeneity should be explored by performing meta-regression and subgroup analysis based on following aspects: year of publication, study type, sample size, detector rows, section thickness, gap, MPR, proportion of serosa-invasive GC subjects, and reference standard. A *Z* test was used to compare the summary estimates of each subgroup, and *P* value  <.05 was thought statistically significant.

Deek funnel plot was used to test for publication bias.^[23]^ All statistical analyses were performed using Stata 14.0.

## 3 Results

### 3.1 Literature searching

**Figure 1:**
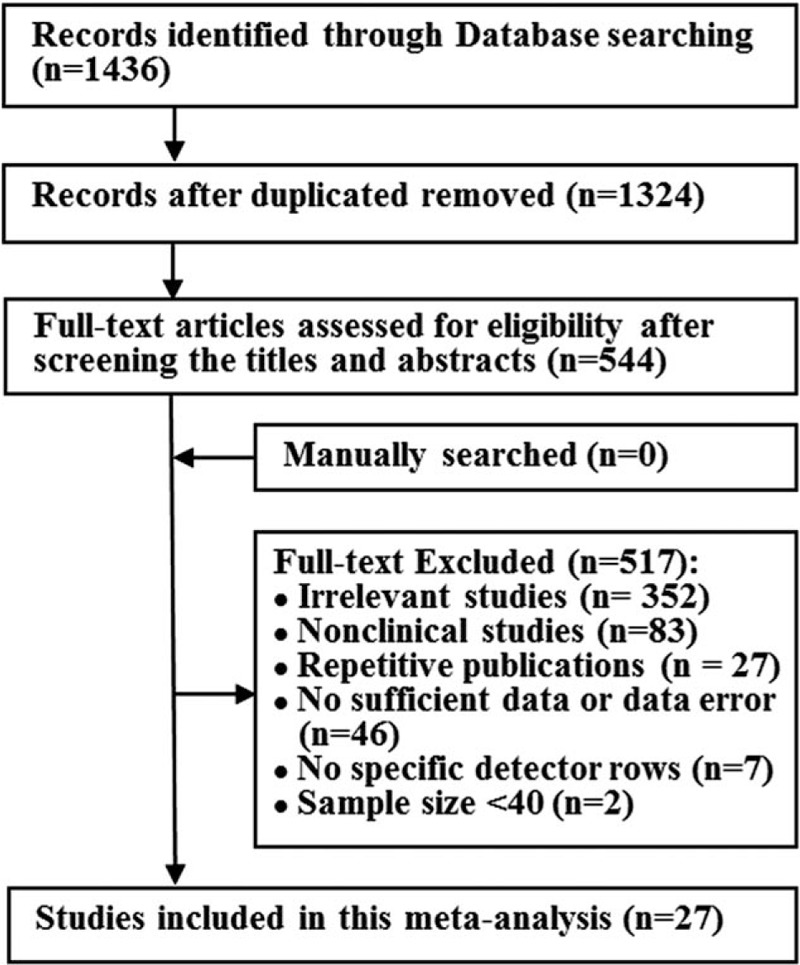
Flow diagram of literature search and study selection.

According to the search strategy, the literature search process was shown in Figure 1. Eventually, 27 studies^[12,13,17,18,24–45]^ were eligible for inclusion after reviewing the full-text.

### 3.2 Study description and patient characteristics

Characteristics of the included studies are presented in Table 1 . The 27 studies had a total of 6519 subjects. Out of these 27, 25 were published in English, 1 in Korean,^[32]^ and 1 in Chinese.^[31]^ About the methodological quality, 19 studies were of high quality (score ≥ 8) and 8 studies were of low quality (score < 8). The proportion of subjects with different T stages was in variety between studies. Then we divided them into 2 groups (A: studies with proportion of serosa-invasive GC subjects ≥50%; B: studies with proportion of serosa-invasive GC subjects <50%). Among the studies, 17 studies were in Group A and 10 studies were in Group B. Besides, 8 studies were investigating the diagnostic performance of MDCT in assessing the lymph node involvement in EGC subjects.

**Table 1:**
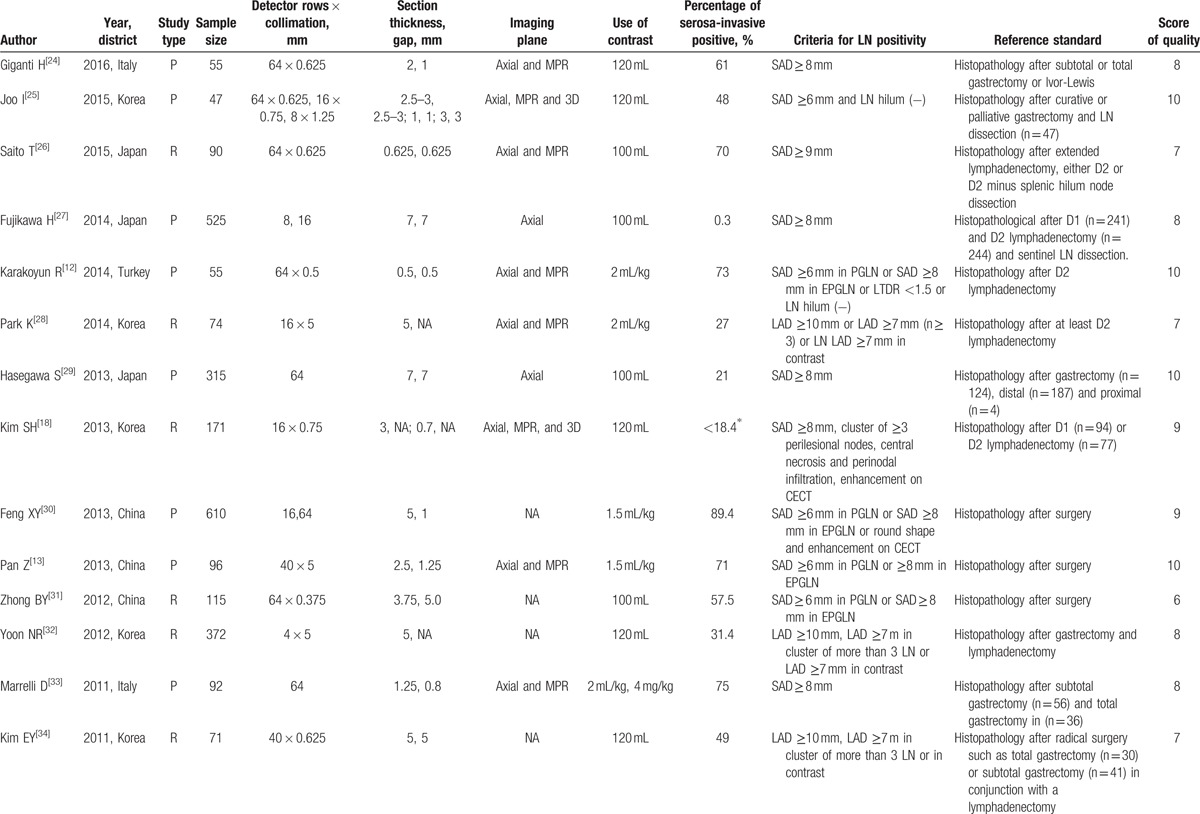
Clinic characteristics of 27 included studies in meta-analysis.

**Table 1 (Continued):**
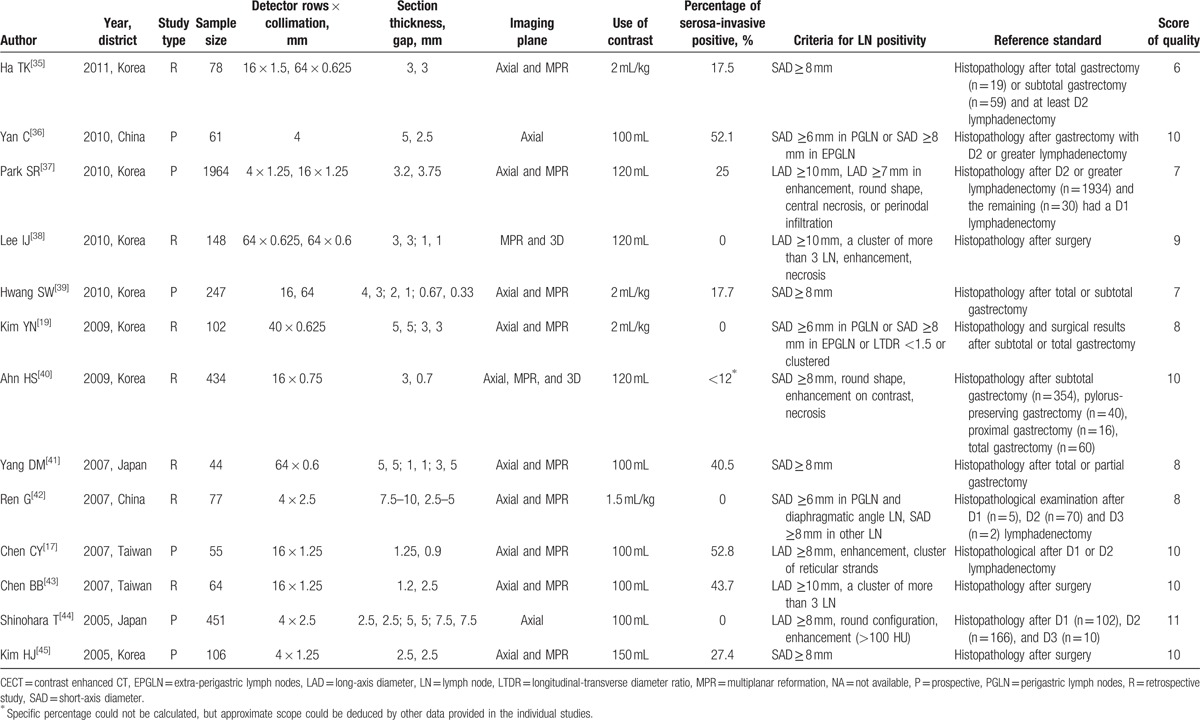
Clinic characteristics of 27 included studies in meta-analysis.

### 3.3 Statistical results

A random effects model was utilized to calculate pooled sensitivity on the basis of statistical heterogeneity (*I*^2^ = 93.75, *P* < .01), and pooled specificity on the basis of statistical heterogeneity (*I*^2^ = 93.08, *P* < .01). The sensitivity and specificity of MDCT ranged from 0.04 (95% CI: 0.01–0.15) to 0.97 (95% CI: 0.87–1.00) and from 0.61 (95% CI: 0.54–0.68) to 1.00 (95% CI: 0.80–1.00), respectively (Table 2); the median sensitivity and specificity were 85.2% and 82.6%, respectively; and the summary sensitivity and summary specificity were 0.67 (95% CI: 0.56–0.77) and 0.86 (95% CI: 0.81–0.90).

**Table 2:**
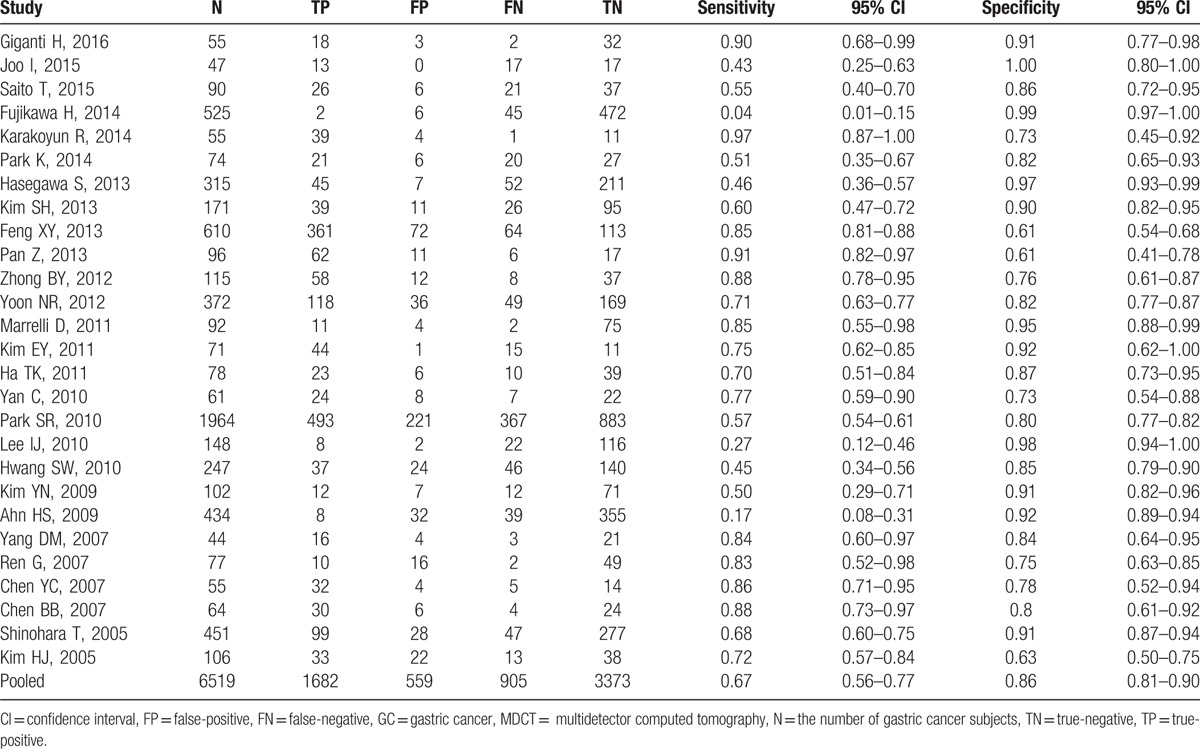
Sensitivity and specificity of MDCT in detection of lymph nodes involvement in preoperative GC subjects in individual studies.

**Figure 2:**
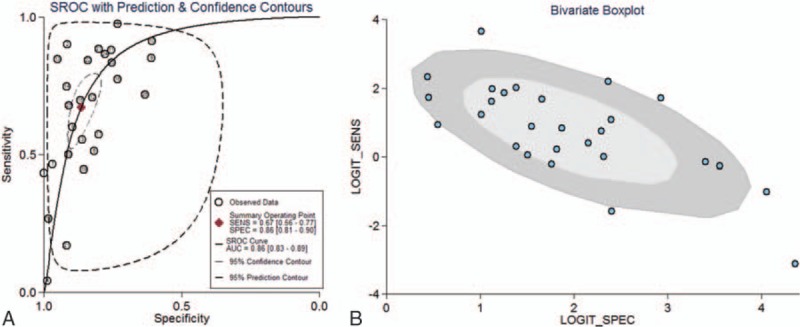
(A) sROC curve of diagnostic performance of MDCT in assessing the preoperative lymph node metastasis of GC subjects; and (B) bivariate boxplot for estimating the threshold variability between the sensitivity and specificity for MDCT in assessing the preoperative lymph node metastasis of GC subjects. GC = gastric cancer, MDCT = multidetector computed tomography, sROC = summery receiver operating characteristic.

Spearman correlation test and bivariate boxplot were used to verify threshold effect. The spearman correlation coefficient was 0.65 (*P* < .001), which suggested that threshold effects existed in this meta-analysis. As shown in Figure 2B, 6 studies stayed the outliers of outer oval in bivariate boxplot, also implying indirect evidence of some threshold variability. Then sROC curve was constructed to summarize the overall diagnostic accuracy. As was seen in Figure 2A, the AUC was 0.86 (95% CI: 0.83–0.89) in total 27 included studies.

### 3.4 Sources of heterogeneity and subgroup analysis

**Figure 3:**
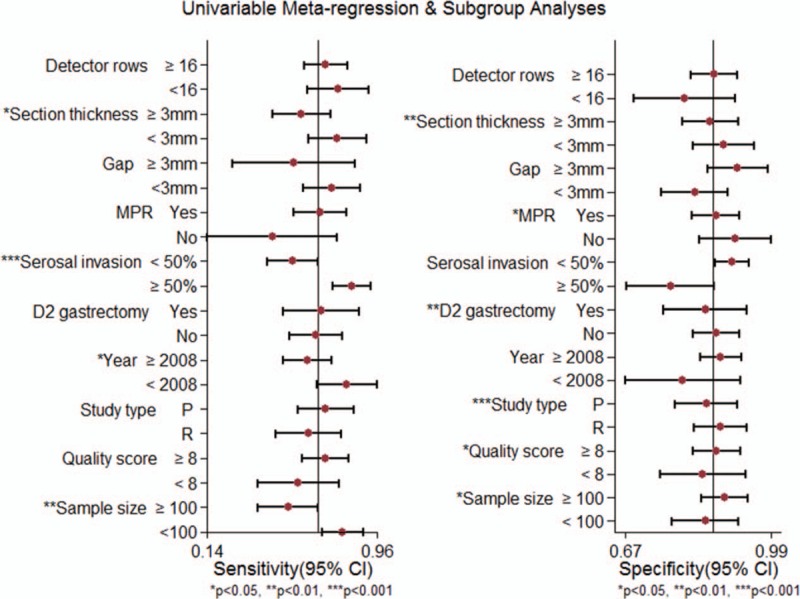
Univariable meta-regression and subgroups analyses of diagnostic performance of MDCT in assessing the preoperative lymph node metastasis of primary GC subjects. GC = gastric cancer, MDCT = multidetector computed tomography, MPR = multiplanar reformation.

To explore the potential sources of heterogeneity, a meta-regression analysis was performed. Of the variables analyzed, section thickness (≥3 or <3 mm), the proportion of serosa-invasive subjects (≥50% or <50%), year of publication (≥2008 or <2008), and sample size (≥100 or <100) showed statistical significance (*P* < .05) in sensitivity analysis, and section thickness (≥3 or <3 mm), MPR (yes or no), D2 gastrectomy (yes or no), study type (prospective or retrospective), score of methodological quality (≥8 or <8), and sample size (≥100 or <100) showed statistical significance (*P* < .05) in specificity analysis (Fig. 3).

Just as Table 3 shows, 26 studies reported the MDCT section thickness. Among them, section thickness is ≥3 mm in 16 studies and <3 mm in the remaining 10 studies. The pooled estimates of MDCT with section thickness ≥3 and <3 mm were 0.59 (95% CI: 0.45–0.74) versus 0.77 (95% CI: 0.63–0.91), *P* = .04, for sensitivity; 0.86 (95% CI: 0.80–0.92) versus 0.89 (95% CI: 0.82–0.95), *P* = .00, for specificity; and 0.83 (95% CI: 0.80–0.86) versus 0.90 (95% CI: 0.87–0.92), *P* = .05, for AUC, respectively. Significant differences were found in sensitivity and specificity analysis between the 2 groups.

**Table 3:**
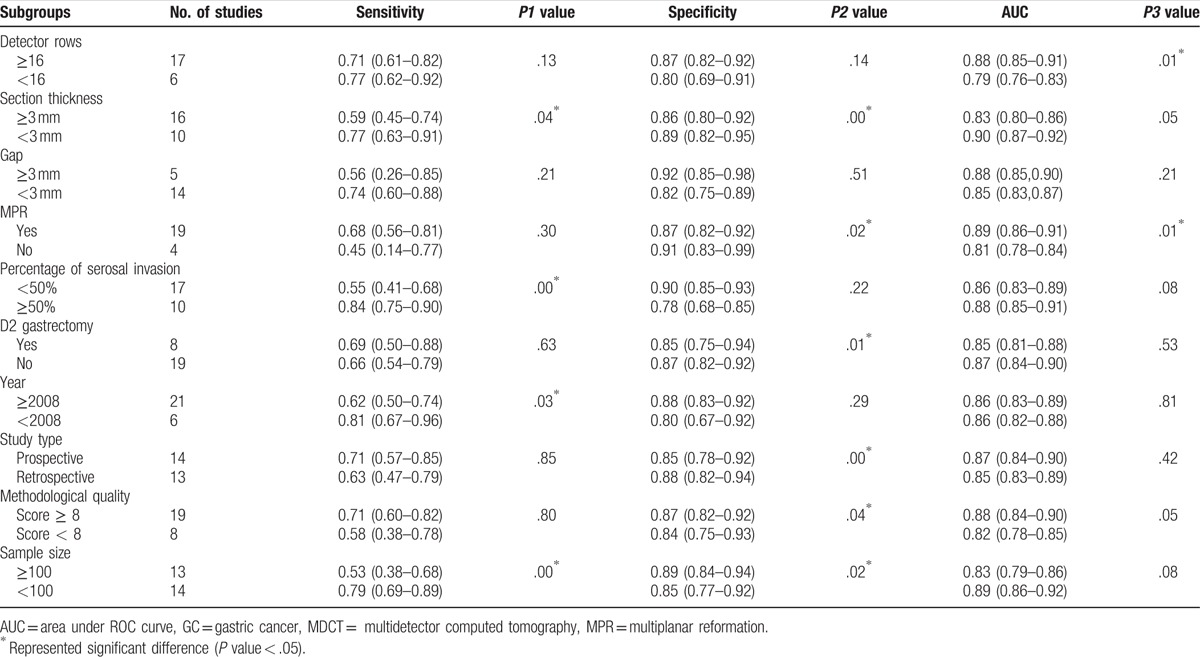
Results of subgroups analysis of diagnostic value for MDCT in detecting lymph node involvement in preoperative GC subjects.

**Figure 4:**
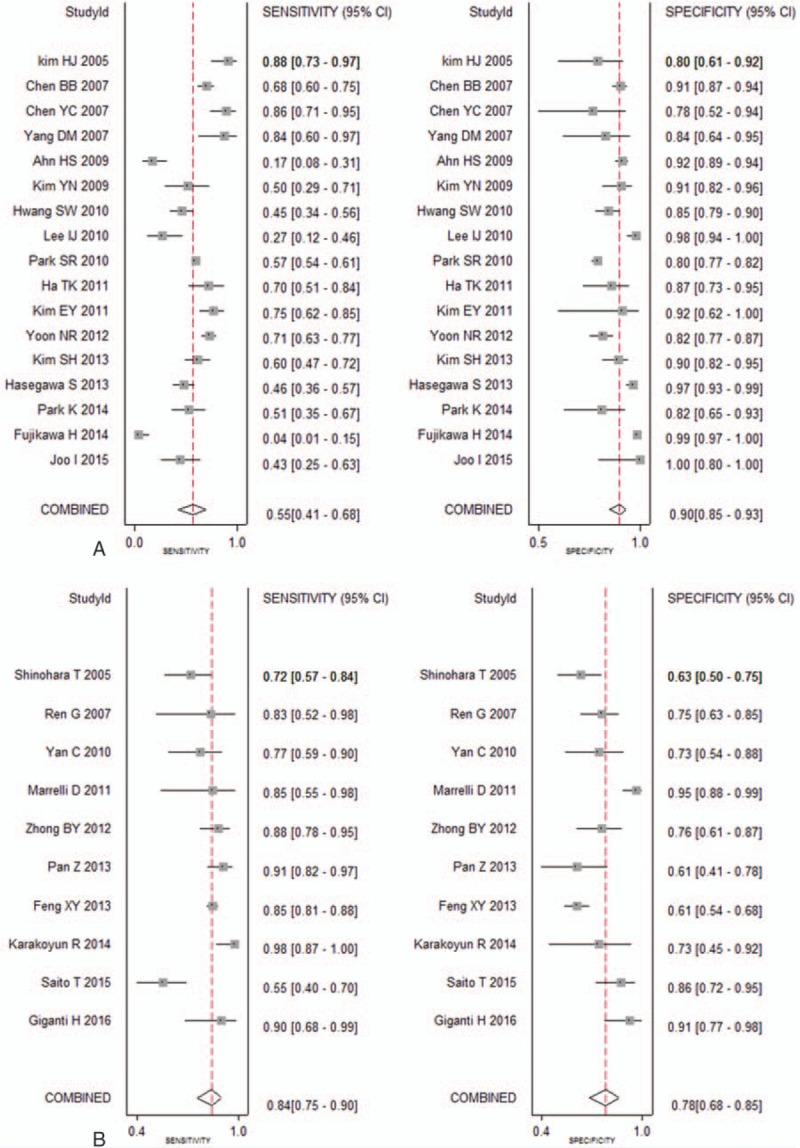
Sensitivities and specificities for MDCT in detecting lymph node metastasis of GC among the studies with percentage of positively serosa-invasive GC <50% (A) and ≥50% (B), respectively. GC = gastric cancer, MDCT = multidetector computed tomography.

The percentage of serosa-invasive GC was calculated in each study, 17 studies in Group A (percentage of serosal invasion ≥50%) and 10 studies in Group B (percentage of serosal invasion <50%) pooled the sensitivities (0.84 [95% CI: 0.75–0.90] vs 0.55 [95% CI: 0.41–0.68], *P* = .00), specificities (0.78 [95% CI: 0.68–0.85] vs 0.90 [95% CI: 0.85–0.93], *P* = .22), and AUCs (0.86 [95% CI: 0.83–0.89] vs 0.88 [95% CI: 0.85–0.91], *P* = .08), respectively (Fig. 4). The sensitivities were statistically different between the 2 groups (Table 3).

**Figure 5:**
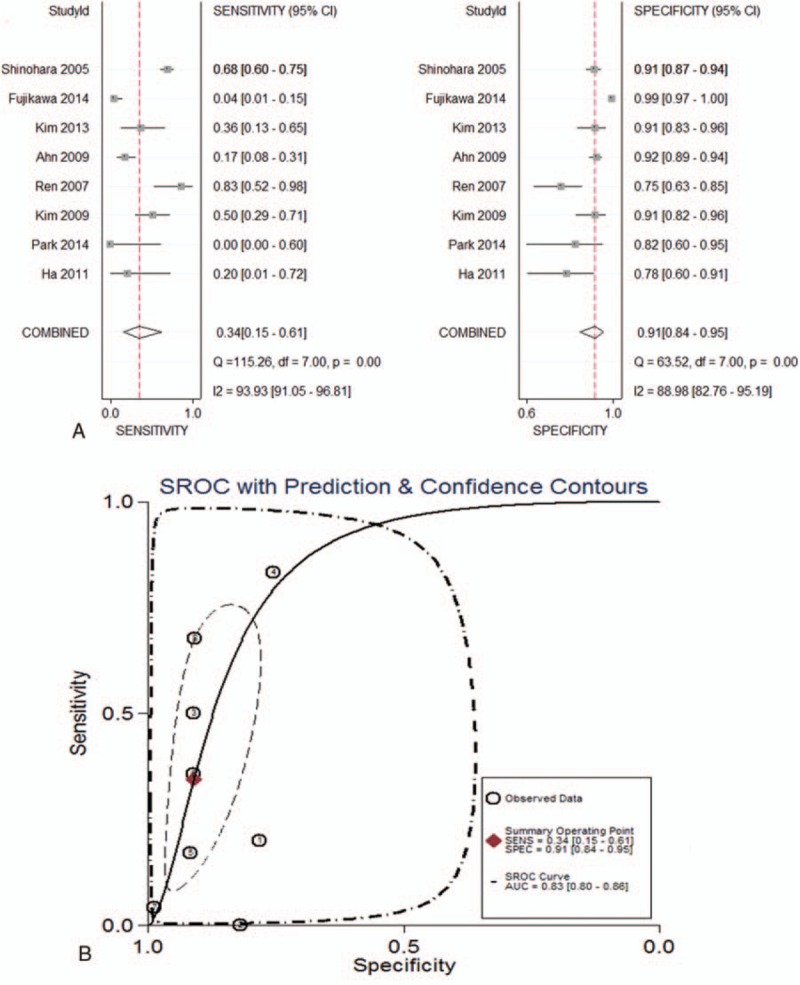
(A) Forest plots of sensitivity and specificity; and (B) summary AUC curve of diagnostic performance of MDCT in detecting preoperative lymph node metastasis of EGC subjects. AUC = area under ROC curve, EGC = early gastric cancer, MDCT = multidetector computed tomography.

In the EGC group, a total of 1086 subjects from 8 studies were selected and the pooled sensitivity, specificity, and AUC for MDCT were 0.34 (95% CI: 0.15–0.61), 0.91 (95% CI: 0.84–0.95), and 0.83 (95% CI: 0.80–0.86), respectively (Fig. 5).

Moreover, subgroup analysis was also performed according to CT detector rows (≥16 or <16), gap (≥3 or <3 mm), MPR (yes or no), D2 gastrectomy (yes or no), year of publication (≥2008 or <2008), study type (prospective or retrospective), sample size (≥100 or <100), and the score of the study quality (≥8 or <8). Details were displayed in Table 3.

### 3.5 Publication bias

**Figure 6:**
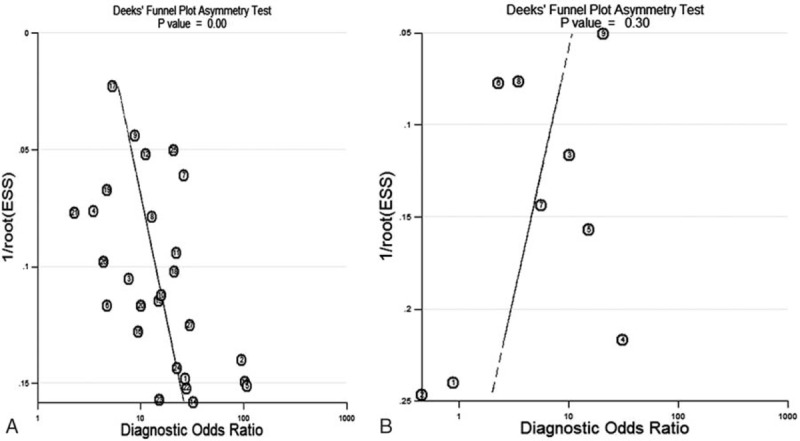
Deek funnel plots for assessing potential publication bias for MDCT in detecting preoperative lymph node metastasis for (A) overall primary GC subjects; and (B) EGC subjects. EGC = early gastric cancer, GC = gastric cancer, MDCT = multidetector computed tomography.

Deek funnel plots suggested asymmetry based on overall GC subjects (*P* = .00) (Fig. 6A), but symmetry based on EGC subjects (*P* = .30) (Fig. 6B), thus providing an evidence of publication bias for overall GC subjects rather than EGC subjects.

## 4 Discussion

LNM was recognized as important to determine the surgical approach and prognosis of GC.^[12]^ MDCT scanning was often performed early in the preoperative evaluation after a diagnosis of GC was made. According to previous studies, its diagnostic performance for assessing the lymph node staging was inconsistent.^[10,12,13]^ In our meta-analysis, the summary sensitivity, specificity, and AUC were 0.67 (95% CI, 0.56–0.77), 0.86 (95% CI, 0.81–0.90), and 0.86 (95% CI, 0.83–0.89) with mild heterogeneities, which resembled the results of Wang et al.^[16]^ The results indicated that the ability of MDCT to stage lymph node (LN) status preoperatively in GC patients was limited because of its low sensitivity.

As MDCT modality continues to evolve, the higher diagnostic accuracy is expected. But confusingly, the recruited studies published after 2008 showed a lower pooled sensitivity than those before 2008 (0.62 [95% CI: 0.50–0.74] vs 0.81 [95% CI: 0.67–0.96], *P* = .03) in subgroup analysis. Obviously, this decreasing sensitivity made it difficult to understand and drew our attention. Afterward we read these articles in depth and found that it was triggered by an unnoticeable variation from participants. In the included studies after 2008, the non-serosa-invasive GC subjects (T1 + T2) accounted for a higher proportion in all participants as a result of the advancements of screening equipment, especially in Japan and Korea.^[18,27–29,35,37]^ So interestingly, afterward we divided these included studies into 2 groups based on the proportion of serosa-invasive GC subjects. The summary sensitivity in Group A (0.84) was significantly higher than Group B (0.55), suggesting that proportion of serosa-invasive GC subjects was an important variable affecting the sensitivity, and MDCT was poor in determining LNM in GC subjects with T1 and T2 stages. Roviello et al^[46]^ and Nasu et al^[47]^ ever concluded that depth of tumor invasion was an independent risk factor of LNM in GC. The early-staged GC had early and microscopic metastatic lymph nodes whose characteristics (including size, shape, necrosis, et al) were not representative; coupled with its low incidence in non-serosa-invasive GC subjects, the involved lymph nodes were not easily detectable by radiologists.^[47]^

In Fareast Asia, including Korea and Japan, a higher proportion of EGC has been commonly detected in routine clinical.^[48,49]^ Among those 27 included studies, we specially screened 8 EGC studies and found the pooled sensitivity was more frustrating: 0.34 (95% CI: 0.15–0.61). Fujikawa et al^[27]^ reported the sensitivity was barely 0.04 (2/47) in clinical T1 staged GC in Japan. Korean scholars Ahn et al^[40]^ also displayed a low sensitivity of 0.17 (8/47) in total 434 EGC subjects. This disappointing sensitivity could be mainly explained by the criteria for positive LNM in our included studies, which was originally developed for advanced gastric cancer (AGC) and might have been too strict for EGC.^[10,28]^ In AGC patients with LNM, large or conglomerated lymph nodes were often seen around the primary lesion.^[50]^ However, EGC, which rarely manifested with LNM, often had early and small metastatic lymph nodes which were not as typical as AGC in MDCT imaging.^[51]^ Microscopic metastases found in normal-sized lymph nodes of EGC subjects were frequently classified as negative because the diameter did not meet the criteria of metastatic LNs, which made accurate N staging more difficult in EGC than in AGC.^[10,52]^ That means MDCT is challenging to appropriately assess lymph node involvement for EGC in clinic. Therefore, in the preoperative evaluation of EGC by MDCT, we need to establish more elaborate and sensitive criteria for LNM to allow the detection of potentially positive lymph nodes.

Similar to the proportion of patients with serosal invasion, the histological subtype might also be one of the potential factors of heterogeneity. A wide variation of histological types (including adenocarcinoma, poorly differentiated adenocarcinoma [PAC], tubular adenocarcinoma [TAC], signet-ring cell carcinoma [SRC], and mucinous adenocarcinoma) existed in the included studies. But so far, few studies have evaluated the value of MDCT in determining the presence of LNM in GC subjects with a single histological type, and subgroup analysis based on pathological type was seldom conducted. So this impact factor cannot be analyzed by meta-regression because it was too mixed and difficult to classify. As both the onset and evolution were different between poorly cohesive carcinoma and intestinal-type carcinoma of GC,^[53]^ the imaging features of metastatic lymph node in MDCT between the 2 histological subtypes might also differ. Of all the included studies, only histological-type-based subgroup analysis by Park et al^[28]^ displayed higher sensitivity, specificity, and accuracy in TAC/PAC than SRC. Meanwhile, contrast-enhanced CT achieved higher sensitivity and accuracy than PET/CT in the detection of regional lymph node involvement in the both histological types.^[28]^

The majority of included studies mainly identified the diagnostic value of MDCT in assessing regional lymph node staging (N staging) of GC, but its role in detecting distant lymph node metastases, which was defined as metastasis reaching or surpassing the terminal node region of the stomach—para-aortic lymph node region—and was classified as M1,^[54,55]^ was seldom explored. Pan et al^[13]^ performed multiphasic 16-slice CT with its scan coverage of parenchymal phase including the entire abdomen to predict distant node metastases, with the results indicating that the preoperative multiphasic CT achieved a sensitivity and specificity of 91% and 97%, respectively. Subsequently, a prospective study^[33]^ reported a satisfying diagnostic performance (85% for sensitivity, 91% for specificity) for determining para-aortic lymph node involvement from GC by MDCT, suggesting MDCT might be a useful tool in diagnosis of distant LNM, and could be helpful to plan surgical approach and neoadjuvant chemotherapy.^[2]^ The aforementioned 2 studies showed an excellent diagnostic value for MDCT in predicting distant LNM. But due to the limited numbers of published articles, the reliability of conclusion needs to be further confirmed.

A sentinel node was defined as the first lymph node that received lymphatic drainage from the primary lesion, and a solitary metastatic lymph node could be considered as a sentinel node in GC.^[56]^ Solitary lymph node metastasis mainly occurred in the perigastric node area,^[57,58]^ but some appeared in extra-perigastric region or even distant area, which was named skip metastasis.^[59,60]^ Sentinel node mapping are recommended in patients with EGC who underwent surgical excision to detect possible skip metastasis.^[61,62]^ A single-skip metastasis located along the middle colic artery was unexpectedly detected in sentinel lymph node biopsy of EGC by Bara's group.^[60]^ Recently, a research showed that LN status (no enlargement of lymph nodes vs swollen lymph nodes) assessed by CT was an independent risk factor for solitary metastatic lymph node, but the specific accuracy in predicting the presence of solitary metastatic lymph node was not mentioned.^[63]^ Kim et al^[64]^ reported on the feasibility of CT lymphography with ethiodized oil for sentinel node mapping in both animal and human studies, and suggested that this updated technique may help make LN dissection minimized in patients with EGC.

MDCT section thickness was another important variable in both sensitivity and specificity. In the subgroups of section thickness ≥3 and <3 mm, the results were 0.59 and 0.77 (*P* = .04) for sensitivity, and 0.86 and 0.89 (*P* < .01) for specificity, respectively. It was not difficult to understand, the thinner slice often had higher sensitivity that could help in finding more subtle lesions. In 2005, Shinohara et al^[44]^ compared the sensitivity and specificity on 3 different slice thicknesses and concluded that they were all dependent on slice thickness of MDCT, and the thinner slice was associated with improved diagnosis value of LNM of GC.

When compared with the separated axial planar MDCT, MDCT with MPR demonstrated no significant difference in sensitivity in our study. In recent studies, N-staging accuracy was not improved by MPR images and 3D display.^[17,45]^ However, a more ameliorative N-staging performance was revealed when MDCT with MPR images was used in AGC cases instead of EGC cases.^[40]^ Therefore, MPR images of MDCT for the evaluation of the preoperative LNM of GC still needs large-sample investigation and analysis to clarity.

Besides, the reference standard was also an important factor that contributed to the heterogeneity of the results. Even though all the positive lymph nodes were referred to gold standard—histopathology—the surgical approaches and the extent of dissection of the lymph node differed according to the preoperative assessment by imaging modalities. So sometimes the positive lymph nodes were preserved because of preoperative misjudgments. In this meta-analysis, only 4 included studies explicitly described that all GC subjects at least adopted D2 lymphadenectomy.^[12,26,28,36]^ Furthermore, the quality of pathological examinations of excised LNs, the skills of the surgeons, and the tumor extensions might also have affected the sensitivities and specificities.

Some potential limitations should be considered in this meta-analysis. First of all, the majority of the included subjects in our study were from Asia. Accordingly, the results might not be helpful in other regions. Second, 14 studies were prospectively designed, but 13 retrospective studies could result in a selection bias in this review. Finally, region-by-region or node-by-node comparison, which might provide other crucial information and more accurate assessments, was not allowed to be implemented in this study.

In conclusion, MDCT tends to be adequate to assess preoperative LNM in serosa-invasive GC, but insufficient for non-serosa-invasive GC, particularly for EGC, owing to its low sensitivity. Proportion of serosa-invasive GC, MDCT section thickness, MPR, and reference standard are the main factors influencing its diagnostic accuracy.
